# Supplementary material for: Empirical methods for controlling false positives and estimating confidence in ChIP-Seq peaks
Source: BMC Bioinformatics. 2008 Dec 5;9:523. doi: 10.1186/1471-2105-9-523 (PMC2628906; doi:10.1186/1471-2105-9-523)
Supplement: Additional File 1 — A variety of html documents from the USeq web site detailing the available applications, their best usage, output file type descriptions, command line menus, etc. [file 1471-2105-9-523-S1.zip › USeqUserGuides/JavaDoc.zip!/JavaDoc/trans/tpmap/class-use/MapFeature.html]

Uses of Class trans.tpmap.MapFeature


|  |  |  |
| --- | --- | --- |
| **Overview** \| **Package** \| **Class** \| **Use** \| **Tree** \| **Deprecated** \| **Index** \| **Help** | |  |
| PREV   NEXT | **FRAMES**    **NO FRAMES**     **All Classes** |  |


---


## **Uses of Class trans.tpmap.MapFeature**

| Packages that use MapFeature | |
| --- | --- |
| **trans.cel** | Apps and methods for manipulating cel files. |
| **trans.tpmap** | For maping, filtering, and calculating statistics about text version bpmap files. |

Uses of MapFeature in trans.cel

| Methods in trans.cel with parameters of type MapFeature | |
| --- | --- |
| `static void` | `CelProcessor.breakAndSaveCelp(java.io.File celpFile, MapFeature[] features)`             Given a float[] celp file and the corresponding MapFeature[] will make a minimal |
| `static void` | `CelProcessor.breakSaveFeatureArray(java.util.ArrayList bpmapInfo, MapFeature[] mapFeatures, java.io.File celpFile)`             Use this method to break and save a celp float[] file into chromosomal components in a directory named after the celpFile minus the .celp extension. |

Constructors in trans.cel with parameters of type MapFeature

`CelMapper(MapFeature[] features,
boolean useMMData,
boolean printStats)`

Uses of MapFeature in trans.tpmap

| Methods in trans.tpmap that return MapFeature | |
| --- | --- |
| `static MapFeature[]` | `ConvertTPMapsToFeatures.convert(java.io.File tpmapFile)`             Converts a text bpmap to a MapFeature[] |

Constructors in trans.tpmap with parameters of type MapFeature

`IntensityFeature(MapFeature feature,
float intensity)`

`MapSplitter(MapFeature[] features,
java.io.File saveDirectory)`
  
          For running in combination with BPMapProcessor

---


|  |  |  |
| --- | --- | --- |
| **Overview** \| **Package** \| **Class** \| **Use** \| **Tree** \| **Deprecated** \| **Index** \| **Help** | |  |
| PREV   NEXT | **FRAMES**    **NO FRAMES**     **All Classes** |  |


---
